# Non-invasive bio-electromagnetic monitoring of cerebrovascular function: a novel conductivity reactivity index (CRx) for optimal cerebral perfusion pressure in acute brain injury models

**DOI:** 10.3389/fbioe.2025.1564510

**Published:** 2025-07-09

**Authors:** Jia Xu, Haochen Li, Feng Wang, Lian Yan, Gui Jin, Mingsheng Chen, Gen Li, Mingxin Qin

**Affiliations:** ^1^ College of Biomedical Engineering and Medical Imaging, Army Medical University, Chongqing, China; ^2^ Department of Medical Engineering, General Hospital of Central Theater Command, Wuhan, Hubei, China; ^3^ School of Pharmacy and Bioengineering, Chongqing University of Technology, Chongqing, China

**Keywords:** magnetic induction phase shift, bio-electromagnetic technology, cerebrovascular function, optimal cerebral perfusion pressure, conductivity reactivity index

## Abstract

**Introduction:**

The pressure reactivity index (PRx) is a key predictor of cerebrovascular function, widely used to guide and optimize therapeutic strategies in patients with acute brain injury. This study investigates a non-invasive bio-electromagnetic technique for monitoring and maintaining cerebrovascular function in a rabbit model of acute brain injury.

**Methods:**

A coaxial parallel double-coil sensor was designed to detect changes in intracranial electromagnetic properties, measured as magnetic induction phase shifts (MIPS), which reflect cerebral blood volume fluctuations. A cerebrovascular function monitoring platform was constructed with this sensor, a vector network analyzer, a LabVIEW software platform, and a physiological signal acquisition device to record the MIPS and arterial blood pressure (ABP). In the animal experiment, a novel cerebrovascular function index Conductivity Reactivity index (CRx), established with MIPS and ABP, was to assess optimal cerebral blood perfusion pressure (CPP) for maintaining the cerebrovascular function in four gradients of CPP in acute brain injury model.

**Results:**

The results found that the CRx (−0.072 ± 0.203) was a significant negative correlation with the PRx (0.223 ± 0.203) (r = −0.447, P = 0.003). Under the optimal CPP determined by the CPP-CRx curve, the mean CRx (0.104 ± 0.170) indicated normal cerebrovascular function, which was significantly different from the other states (CRx = −0.127 ± 0.061, p = 0.009).

**Discussion:**

The study demonstrated that CRx has potential to reflect cerebrovascular function dynamics and assess optimal CPP, demonstrating the potential of bio-electromagnetic technology as a noninvasive indicator for monitoring cerebrovascular function.

## 1 Introduction

The Pressure Reactivity Index (PRx) is a cerebrovascular compliance indicator, derived from the relationship between the invasive intracranial pressure (ICP) and cerebral perfusion pressure (CPP) (Czosnyka et al., 1997). It plays a pivotal role in the maintenance of cerebrovascular function for patients with acute brain injury (ABI), reducing secondary brain injury caused by abnormal cerebral blood flow perfusion (Feigin et al., 2020; Zeiler et al., 2022). Maintaining optimal cerebrovascular function is important for improving patient outcomes, reducing disability, and lowering mortality rates (Needham et al., 2017). Clinical, real-time monitoring of PRx at the bedside can assess the severity of brain injury and guide management of cerebral blood perfusion for patients with ABI (Steiner et al., 2002).

Building on the relationship between PRx and cerebrovascular function, the concept of maintaining an optimal cerebral perfusion pressure (CPPopt) has emerged as a strategy to achieve better outcomes in patients with severe brain injury (Weiss et al., 2022; Dias et al., 2015). The conventional method for determining CPPopt relies on the CPP–PRx curve (Zeiler et al., 2022). However, non-invasive assessments of CPPopt for patients with mild brain injury are still under investigation. Near-infrared spectroscopy (NIRS) provides a non-invasive approach to assess CPPopt using the Cerebral Oximetry Index (COx) (Zweifel et al., 2010; Hoiland et al., 2020; Gaasch et al., 2018). Yet, the correlation between COx and the PRx can be influenced by factors such as inadequate light intensity and significant disparities in detection signals between the two brain hemispheres (Rivera-Lara et al., 2019; Diedler et al., 2011; Smielewski et al., 2010). Transcranial Doppler (TCD), another non-invasive method, can determine CPPopt based on the mean flow velocity index (Mx) when it incorporates robotic-assisted units for probe automatic adjustment (Zeiler et al., 2018). However, these robotic-assisted units are not universally used due to their high cost, which limits their widespread application in assessing CPPopt. Additionally, the Mx index is currently limited to monitoring blood flow velocity in specific larger vessels, such as the Middle Cerebral Artery, and is not sufficient for monitoring the cerebrovascular function of the global brain.

Recently, advances in bio-electromagnetic technologies have shown promise in obtaining non-invasive monitoring indicators of cerebrovascular function. Techniques such as rheoencephalography (Bodo et al., 2004), transocular impedance (Tiba et al., 2022), and electromagnetic coupling (Gong et al., 2024) have been explored. However, these methods are limited to detecting local cerebral blood perfusion in regions where an excitation current is applied (Tiba et al., 2017). For deep cerebral blood flow measurements, electrode placement near the target area is often required, making the procedure invasive (Szabo et al., 2024). Magnetic induction technology, a non-contact bioelectromagnetic detection technique, reflects changes in cerebral blood volume conductivity induced by fluctuations in blood perfusion (Oziel et al., 2016). In our previous research, the conductivity reactivity index (CRx), derived from the phase shift of the magnetic induction detection signal, was identified as a reliable indicator for distinguishing cerebrovascular functional impairments following ischemia (Xu et al., 2023). Unlike contact-based methods, CRx provides a non-contact, whole-brain assessment approach for cerebrovascular function, offering greater convenience and ease of use. Although these bio-electromagnetic detection technologies have demonstrated the capability for non-invasive cerebrovascular monitoring, their effectiveness in determining CPPopt and guiding cerebral blood perfusion management remains unproven.

This study aims to evaluate the feasibility of non-invasive bio-electromagnetic detection technology for determining CPPopt and its potential role in individualized cerebral blood perfusion management through an animal experimental study.

## 2 Materials and methods

### 2.1 Bio-electromagnetic detection principle

Based on the principle of the two-port vector network detection (Li et al., 2019), the electromagnetic transmitted the brain will be modulated by the fluctuations of the brain’s electromagnetic properties. The transmission coefficient (TC) is defined as the voltage ratio of the transmitted signal to the incident signal, as shown in Equation 1.

$$TC=\frac{V_{Transmission}}{V_{Incident}}=\tau ∠\varphi$$ (1)

Here, *τ* represents the amplitude of the transmission coefficient, and *φ* is the phase shift between the transmitted and incident signals. This phase shift is termed the Magnetic Induction Phase Shift (MIPS) in this context.

**FIGURE 1:**
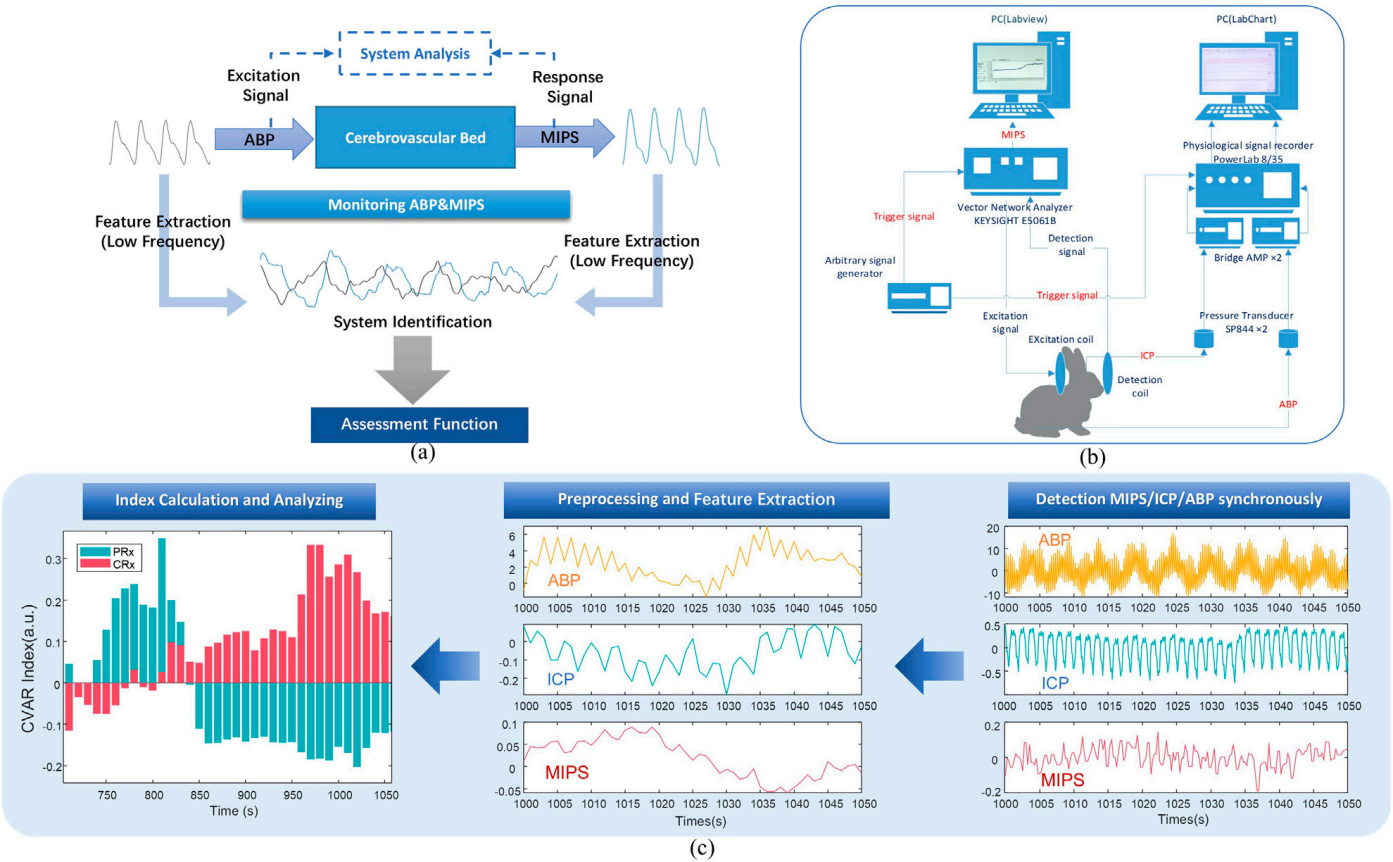
Block diagram of the cerebrovascular function detection principle and experimental system. **(a)** The systematic evaluation and analysis principle of cerebrovascular function. **(b)** Block diagram of experimental system. **(c)** The process of acquiring the CRx and PRx.

The MIPS is directly associated with the electromagnetic properties of the brain’s bulk tissues. Intracranial tissues, including cerebrospinal fluid (CSF), cerebral blood flow (CBF), gray matter, and white matter, exhibit significant differences in their electromagnetic properties (Gabriel et al., 1996). Changes in cerebral blood perfusion pressure result in alterations in cerebral blood volume, which subsequently modify the volumetric proportions of these intracranial tissues. These changes lead to variations in the global electromagnetic properties of the brain and influence the phase of the TC. Consequently, variations in MIPS can serve as a reliable indicator of cerebral blood perfusion status. By treating peripheral arterial pressure as the input excitation to the cerebrovascular bed and MIPS as the response, analyzing the low-frequency feature changes between them provides a functional assessment of the cerebrovascular system, as illustrated in Figure 1a.

### 2.2 Coaxial parallel double-coil sensor

**FIGURE 2:**
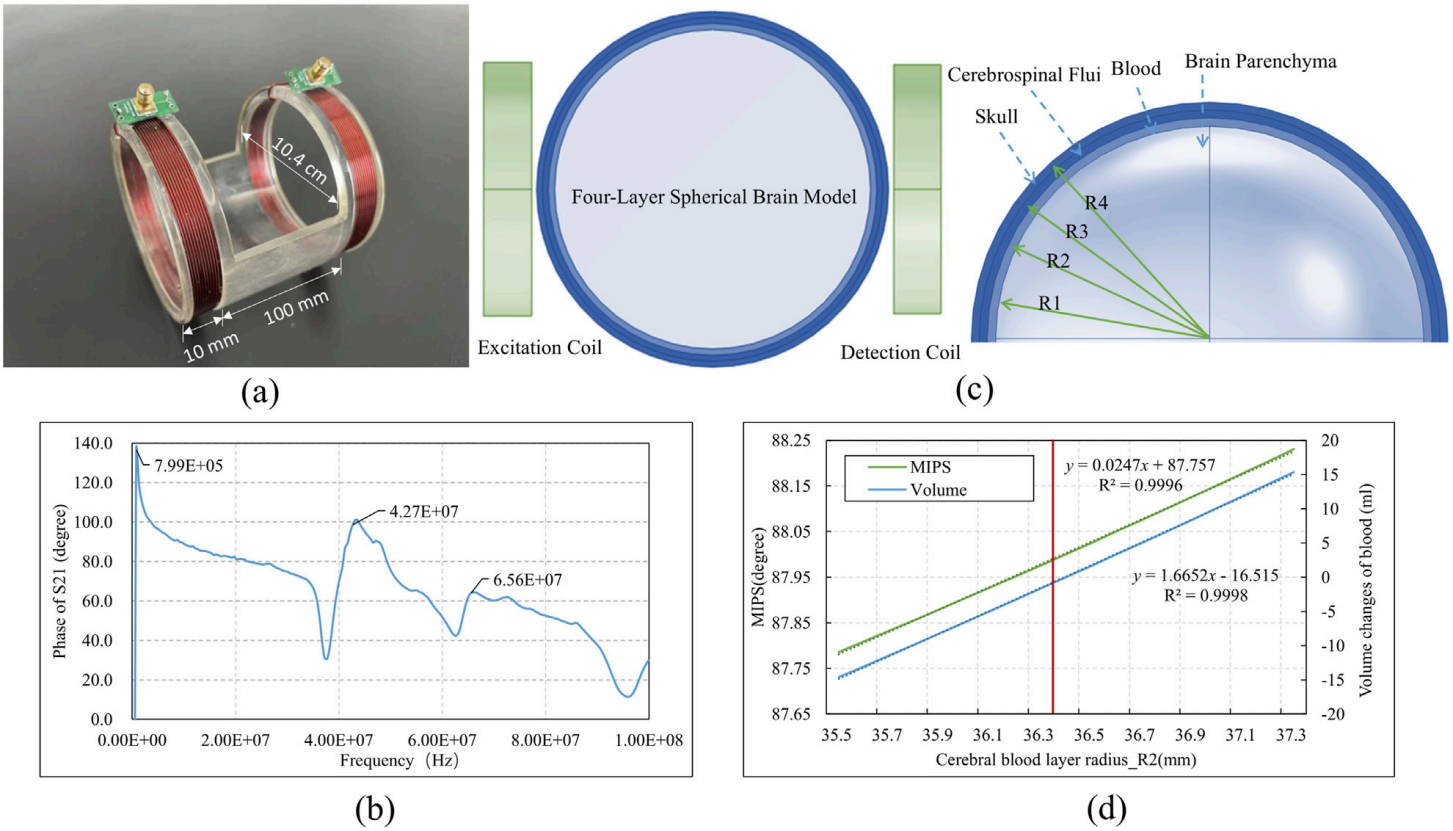
The structure and simulation of the coaxial parallel double-coil sensor **(a)** Sensor dimensions **(b)** Sweeping phase of S21 from 300 kHz to 100 MHz **(c)**Sensor simulation with brain model **(d)** Simulation result.

This study utilized a coaxial parallel double-coil sensor to capture response in MIPS, addressing the need for whole-brain measurements and advancing further multi-frequency research. As shown in Figure 2a, two coils were wound from AWG 32 copper wire of the 1 mm diameter, one serving as the excitation coil and the other as the detection coil, with 10 turns for both. The radius of the coils and the distance between them were adapted to the size of the rabbit brain being measured. The radius of each coil is 5.2 cm and the coils spaced 10 cm apart from each other. The frequency sweeping results of the coil are presented in Figure 2b, revealing three phase peaks at 800 kHz, 42.7 MHz, and 65.6 MHz. Additionally, considering the skin depth formula $\delta =1/\sqrt{\pi f\mu \sigma }$ (Hayt and Buck, 2012), 42 MHz offers a suitable depth for penetrating and covering the entire rabbit brain (∼8 cm in diameter), ensuring effective detection. It also demonstrated better phase stability (0.050°/h) than 65.6 MHz (0.330°/h). Based on above reason in this study, a single frequency point of 42 MHz was selected for MIPS detection.

To assess the coil’s ability to detect changes in cerebral blood volume near the 42 MHz frequency, an electromagnetic simulation was conducted using a simplified four-layer spherical brain model with a diameter of 8 cm. The model consists of four concentric layers: the skull, CSF, brain blood, and brain parenchyma. The radii of these layers, determined by the proportional volumes of brain parenchyma (80%), brain blood (10%), and CSF (10%), were as follows: R1 = 35 mm, R2 = 36.4 mm, R3 = 37.7 mm, and R4 = 39 mm. The dielectric constants and conductivities of each layer, as presented in Table 1, demonstrate that variations in brain blood and CSF primarily influence conductivity within the model. The simulated coaxial parallel coils were configured to match the model’s dimensions, with a radius of 4.4 cm, 10 turns, an 8 cm separation, and a wire diameter of 1 mm. These coils were positioned on either side of the model, as shown in Figure 2c, with the left coil designated for excitation and the right coil for detection. The experiment adjusted the R2 value to modulate cerebral blood volume: increasing R2 resulted in an enlarged brain blood volume and a corresponding decrease in CSF volume, while decreasing R2 led to reduced brain blood volume and increased CSF volume. The MIPS between the two coils was measured to track these changes. The simulation was conducted using the finite element analysis software COMSOL Multiphysics (version 6.0, COMSOL AB, Sweden).

**TABLE 1:** Electromagnetic Property parameter settings for the four-layer spherical brain model.

Tissue	Dielectric constant	Conductivity (S/m)
Brain parenchyma	98.2	0.317
Brain blood	103.0	1.180
CSF	103.0	2.030
Skull	18.9	0.055

In Figure 2d, the red line indicated the initial R2 value. When R2 increased from 36.4 mm to 37.3 mm, the blood volume increment ranged from 0 to 15.36 mL, and the MIPS shifted from 88.00° to 88.23°, corresponding to an MIPS change of approximately 0.015°/ml due to the blood volume increase. Conversely, a decrease in blood volume resulted in a reduction in MIPS.

Using a physical model with manually injected saline at varying volumes, we determined the system sensitivity operating on 42 MHz to be 0.180°/ml, which shows an improvement compared to our previous study at 6 MHz (Xu et al., 2023). Additionally, the system exhibited good linearity (R^2^ = 0.9968) across different volume measurements.

### 2.3 Experimental plant

**FIGURE 3:**
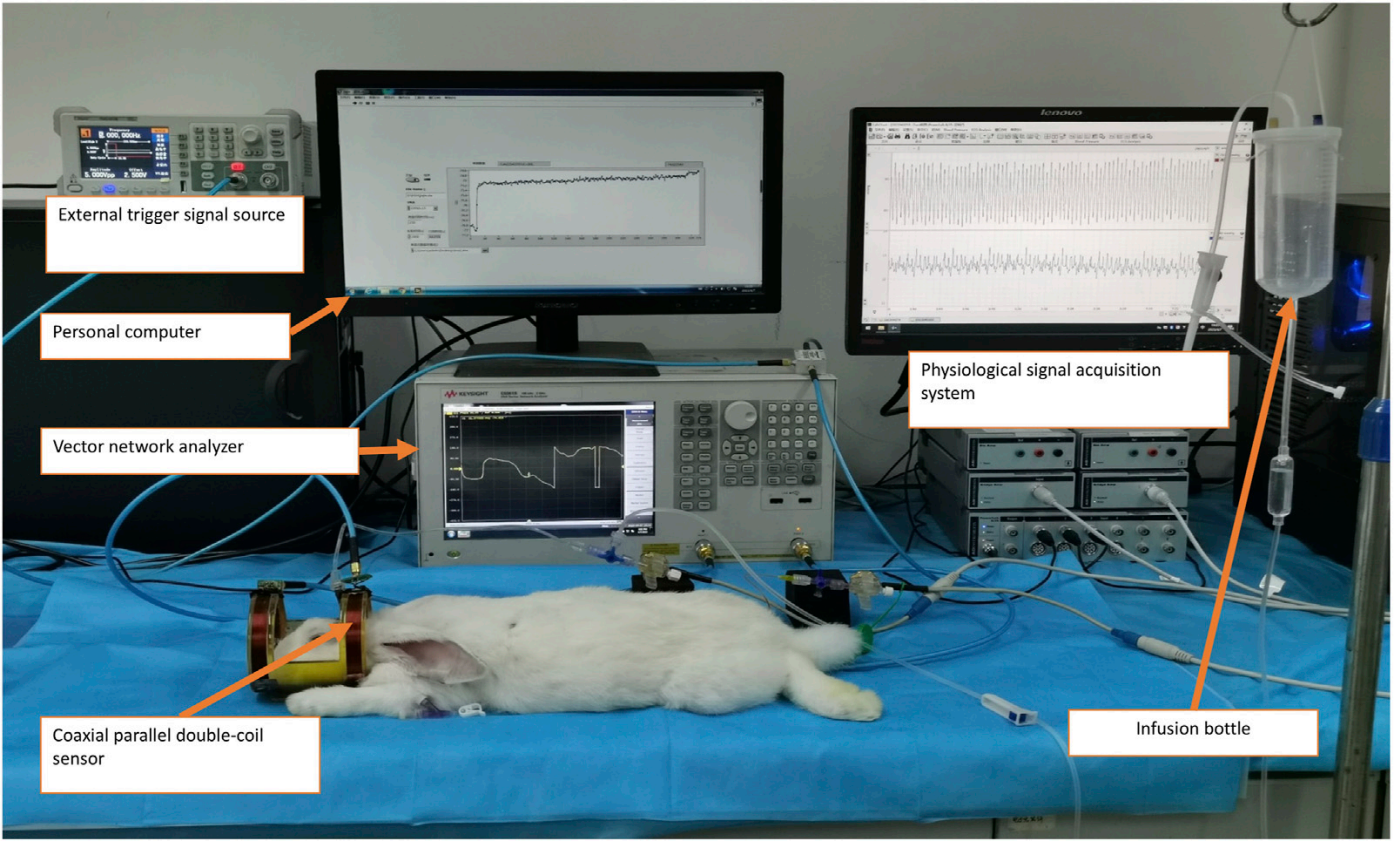
Experimental measurement of the cerebrovascular function detection system on a rabbit with intracranial hypertension.

The animal experimental platform for cerebral vascular function monitoring, as illustrated in Figure 3, utilizes the aforementioned sensor and consists primarily of two key components: a single-frequency MIPS detection system and a physiological signal acquisition device.

The MIPS detection system includes a vector network analyzer (Agilent E5061B, Keysight) and a personal computer (PC) running a custom single-frequency phase shift extraction program developed in LabVIEW software (version 2018, National Instruments, United States). The MIPS signal, representing the phase shift of S21 at 42 MHz, is captured by the sensor and extracted from the vector network analyzer through the phase shift extraction program. Concurrently, ICP and ABP are acquired through the physiological signal acquisition system. This system comprises a PowerLab 8/35 acquisition instrument (AD Instruments, Australia), two pressure sensors (SP844), and two bridge amplifiers. To ensure synchronization of the ABP, ICP, and MIPS signals, an external trigger signal generator produces a square wave to initiate synchronized data acquisition across the systems.

### 2.4 Animal model

The animal experiments conducted with this platform were reviewed and granted approval by the Animal Experiment Ethics Committee of the Army Medical University, with the approval number AMUWEC20230300, on March 3, 2023. Ten male New Zealand white rabbits, weighing between 2 and 2.5 kg, were randomly obtained from the university’s Animal Experiment Center and assigned identification numbers from No.1 to No.10. All experiments were conducted under environmentally controlled laboratory conditions, with ambient temperature maintained at 25°C ± 0.5°C and relative humidity at 65% ± 2%, using an air conditioning system and a humidifier to ensure stability during signal acquisition.

An animal model of intracranial hypertension was established to assess the CPPopt based on the CRx/PRx. After anesthesia, tracheotomy was performed on the rabbits, followed by intubation for ventilation. Subsequently, an arterial catheter was inserted into the right femoral artery and connected to a physiological pressure sensor (SP844) for continuous monitoring of arterial blood pressure (ABP).

A 1.6 mm probe was then inserted into lateral ventricle of the rabbit’s brain to monitor ICP. The probe was connected to a T-joint with a silicone tube. The other ports of the T-joint were linked to another physiological pressure sensor (SP844) in the physiological signal acquisition device and an infusion bottle containing Ringer’s solution. The pressure in the lateral ventricle was maintained at four hydrostatic levels of approximately 10 mmHg, 20 mmHg, 40 mmHg, and 60 mmHg by the hydraulic pressure of the infusion bottle to obtain four CPP gradients.

### 2.5 Data acquisition

The experimental platform was connected as the block diagram shown in Figure 1b. Subsequently, the acquisition parameters of the platform were configured. For the MIPS detection system, the parameters were set to a sweep frequency range of 300 kHz to 100 MHz, an intermediate frequency bandwidth of 30 kHz, and the detection channel was set to S21. The system was further set to operate in external trigger mode, activated on the rising edge, to extract the single-frequency signal at 42 MHz with a sampling interval of 0.25 s.

Following the animal model preparation, rabbits were monitored for 30 min at an ICP of 10 mmHg. Then the ICP was increased to 20 mmHg, and after stabilization, they were monitored for an additional 30 min. This procedure was repeated with ICP level of 40 mmHg and 60 mmHg, each followed by a 30-min monitoring period. The acquisition data were stored and subsequently analyzed offline to calculate the CRx index and perform statistical analyses.

### 2.6 Data processing

The ABP, ICP, and MIPS signals were manually processed to remove motion artifacts, resulting in a total of 41 datasets from 10 rabbits. Sample No.8 exhibited fluctuations along with a simultaneous decrease in ICP and ABP at the 10 mmHg level, necessitating a repeated measurement at that level. The CPP was derived from the ABP and ICP values. For each ICP level, the mean and standard deviation of ABP, ICP, MIPS, and CPP were calculated and analyzed.

Before the calculation of CRx and PRx, the aforementioned physiological signals were preprocessed as illustrated in Figure 1c. Baseline drift was removed from all signals, which were then down-sampled to 1 Hz and smoothed over 10 data points. For each 30-min recording, the data were averaged over 10-point intervals. Slow oscillation components (0.005 to 0.05 Hz) were extracted with a 3 dB Butterworth band-pass filter. Using the preprocessed signals, a moving Pearson correlation coefficient was calculated with a 5-min window updated every 10 s to generate the PRx and CRx series (Xu et al., 2023). The CRx was derived from the Pearson correlation between MIPS and ABP (Equation 2), while the PRx was derived from the correlation between ICP and ABP (Equation 3).

$$CRx=r\_s\left(ABP\;and\;MIPS\;over\;300\;points\right)$$ (2)

$$PRx=r\_s\left(ABP\;and\;ICP\;over\;300\;points\right)$$ (3)

Data preprocessing and CRx and PRx calculation were implemented through a custom program in MATLAB (version R2014, MathWorks, United States) software.

The mean PRx curves were plotted with CPP as the horizontal axis and then smoothed using the Bezier algorithm. In the CPP-PRx curve, optimal CPP (CPPopt) was identified as the lowest position on the U-shape curve (Gabriel et al., 1996). The CPP-CRx curves were obtained in the same manner. The CPPopt was attempted to be obtained from the CPP-CRx curve in relation to the CPP-PRx relationship.

### 2.7 Statistical analysis

To compare the PRx and CRx, the mean and standard deviation of each index were analyzed, along with the correlation between them. A linear regression analysis was conducted to determine the relationship between the two indexes. The Bland–Altman analyses and intraclass correlation coefficient (ICC) assessments were conducted to more comprehensively evaluate the agreement between CRx and PRx. To clarify the applicable scope of CRx, analyses were conducted based on the full dataset and a CPP >40 mmHg subset that excluded conditions of severe hypoperfusion, used of–CRx corrects for the known physiological inverse relationship with PRx.

In addition to human ABI studies (Sorrentino et al., 2012; Zeiler et al., 2020), animal models (Beqiri et al., 2021; Klein et al., 2024) have also reported that PRx >0.25–0.3 correlates with impaired autoregulation and poor neurological outcomes. Take PRx >0.25 as the threshold for CVAR injury, using the linear regression model, calculated the injury threshold of CRx.

To further investigate the optimal threshold for CRx in identifying impaired cerebrovascular autoregulation, we performed a threshold classification analysis across a series of reference PRx cutoffs. Specifically, we generated binary impairment labels at seven PRx thresholds (from 0.00 to 0.30, interval 0.05), treating values above each threshold as impaired. For each threshold, a ROC analysis was conducted using–CRx as the predictive variable. The optimal CRx cutoff was determined using the Youden index. Area under the curve (AUC), sensitivity, and specificity were computed for each PRx reference level to evaluate diagnostic performance and consistency of CRx behavior. Considering the influence of CPP, the classified samples are subsets with CPP greater than 40 mmhg.

To investigate the potential application of CRx in cerebral blood perfusion management, differences between the index values near CPPopt and those at other CPP levels were analyzed. The indexes were categorized into two groups based on whether their values were at CPPopt. Each rabbit’s CRx and PRx values at the optimal CPP (CPPopt) were compared to the mean values at the remaining non-CPPopt levels. A non-parametric paired test, using the Wilcoxon signed-rank test, was conducted to compare CPPopt and non-CPPopt states within each subject (n = 8). To account for within-subject variability and confirm robustness, linear mixed-effects (LME) models were applied with CPP condition (CPPopt vs. non-CPPopt) as a fixed effect and rabbit ID as a random effect. To confirm that this exclusion did not bias the PRx–CRx association after excluded No. 6 and No. 7, we conducted the Correlation analysis between PRx and CRx on the 8 sample with effect size analysis using Cohen’s q yielded. Statistical analyses were performed using SPSS (version 25, IBM, United States), Origin (version 2017, OriginLab, United States) and Python (version 3.10.12, Python Software Foundation, United States).

## 3 Results

### 3.1 Physiological parameters at various ICP stages

**FIGURE 4:**
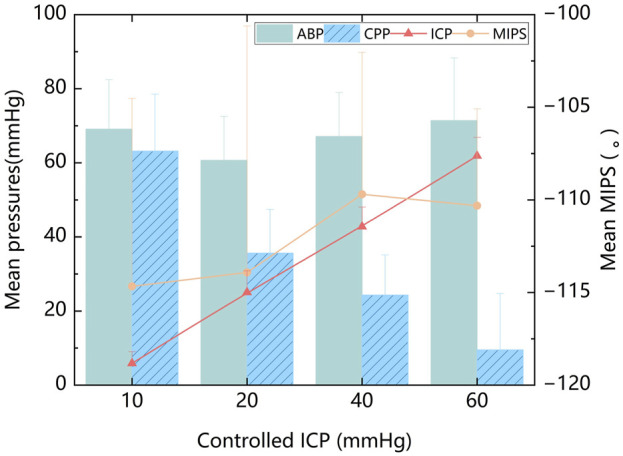
The mean ABP, ICP, MIPS, and CPP at four controlled ICP levels.

The mean values of ABP, ICP, MIPS, and CPP were calculated from all experimental samples. Across all ICP states, the mean ABP was 67.1 ± 14.1 mmHg. The mean ICP was 33.2 ± 21.1 mmHg. The mean MIPS was 112.2 ± 10.2°. The mean CPP was 33.9 ± 24.3 mmHg. When ICP increased regularly with relatively stable ABP, the animal model exhibited gradient changes in CPP, as shown in Figure 4. The MIPS, however, did not exhibit a consistent linear relationship with ICP. When the ICP was below 40 mmHg, the MIPS increased with the ICP, and inversely changed with ICP between 40 and 60 mmHg. This phenomenon may be attributed to changes in intracranial tissue volume altering the MIPS, while the compensation mechanism of cerebrospinal fluid did not vary linearly across different ICP levels. There was no significant correlation between the mean values of ABP and MIPS (P = 0.260) or between ABP and ICP (P = 0.595).

### 3.2 Data preprocessing results

**FIGURE 5:**
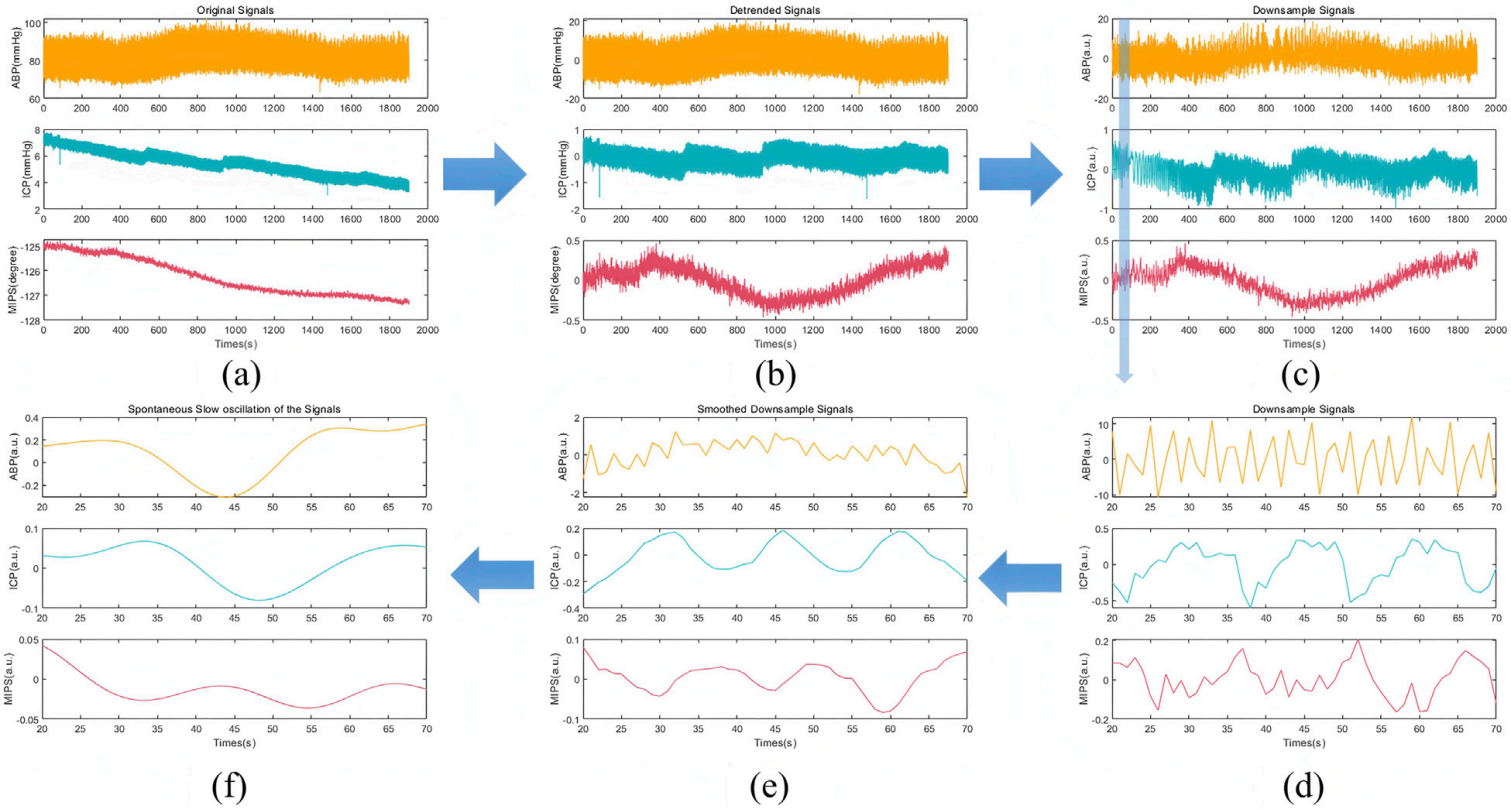
The preprocessing process of ABP, ICP and MIPS signals in rabbit Exp.3 **(a)** The original signals **(b)** The removed baseline signals **(c)** The down sampling signals (1 Hz) **(d)** The enlarged shadow portion of the down sampling signals **(e)** The smoothed down sampling signals **(f)** The slow oscillation components (0.005 to 0.05 Hz).

The preprocessing process of ABP, ICP and MIPS signals was depicted in Figure 5. In the Figure 5e, it was observable that the MIPS signal changed inversely with the ICP signal. After baseline removal, down sampling, and smoothing, the slow oscillation components (0.005 to 0.05 Hz) were extracted for calculation of CRx and PRx. The slow oscillation component of ICP, as seen in Figure 5f, changed with the spontaneous oscillation of ABP, suggesting potential cerebrovascular injury at that time. Conversely, the MIPS showed an inverse association with the slow oscillation component of ABP, which also suggested potential vascular dysfunction.

### 3.3 The relationship between CRx and PRx at various ICP levels

**FIGURE 6:**
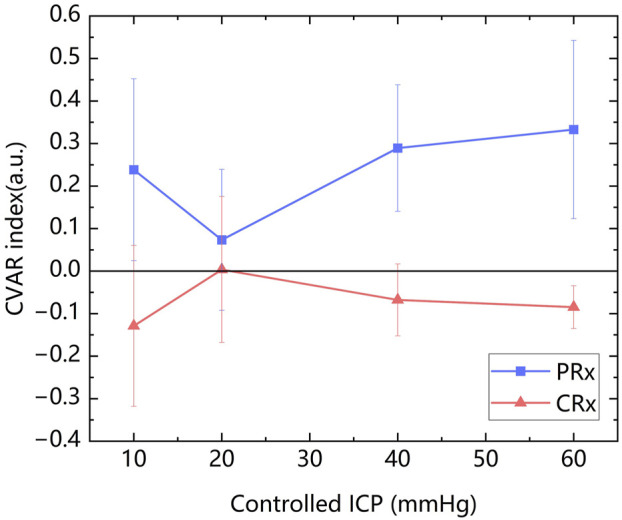
The changes in the PRx and the CRx with the increase of ICP.

The mean values of PRx (0.223 ± 0.203) and CRx (−0.072 ± 0.203) were calculated. The mean values of PRx and CRx of ten rabbits at each controlled ICP level were presented in Figure 6. It can be observed that CRx changed inversely with PRx, peaking at an ICP level of 20 mmHg, while PRx reached its lowest value at this stage, indicating minimal cerebrovascular function injury. PRx and CRx exhibited a significant negative correlation (r = −0.447, P = 0.003). Due to the varying ICP-MIPS relationship, a correlation analysis of the two indexes was performed for the first two ICP states. There was a higher negative correlation between the PRx and the CRx (r = −0.638, P =0.002) in the first two ICP states.

The linear regression analysis revealed a linear relationship of the two indexes: CRx = −0.312PRx - 0.002 (R^2^ = 0.200, P < 0.005). According to this linear relationship, an injury threshold of approximately −0.08 for CRx was determined based on a severe cerebrovascular function injury threshold of 0.25 for PRx (Beqiri et al., 2021; Klein et al., 2024).

**FIGURE 7:**
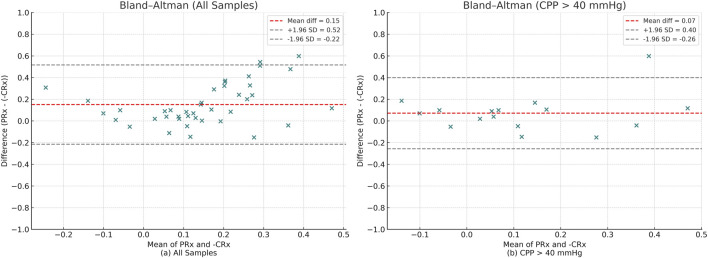
Bland–Altman plots comparing PRx and–CRx values. Red dashed lines represent the mean difference, and gray dashed lines represent the 95% limits of agreement (±1.96 SD). **(a)** Analysis across all samples. **(b)** Analysis restricted to samples with CPP >40 mmHg.

Bland–Altman analysis of the full dataset demonstrated poor agreement between PRx and inverted CRx, with a mean difference of 0.15 and wide limits of agreement (−0.23 to 0.52) (Figure 7a). This indicates that across all conditions, PRx and CRx are not directly interchangeable. When restricting the analysis to measurements with CPP >40 mmHg, the agreement between PRx and inverted CRx improved. The mean difference decreased to 0.07, and the limits of agreement narrowed (−0.26 to 0.40), suggesting moderate agreement under higher cerebral perfusion pressures (Figure 7b).

The ICC analysis using PRx and inverted CRx values for the full dataset showed poor consistency: ICC = 0.205 (poor). However, for CPP >40 mmHg subset ICC values improved significantly: ICC = 0.583(Moderate). This indicates a moderate level of agreement, when the CPP was not in an extremely low state.

**FIGURE 8:**
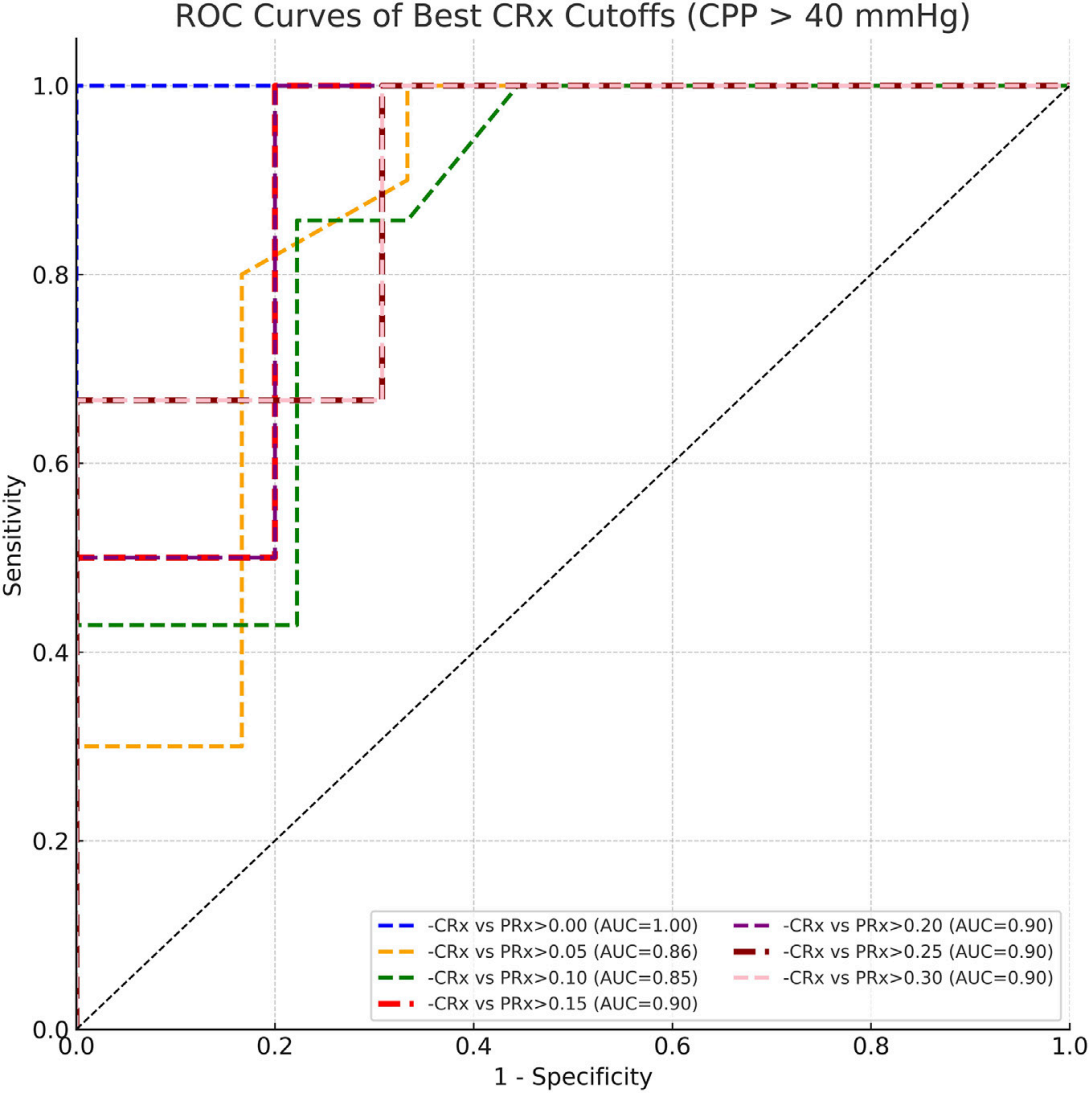
ROC curves comparing CRx cutoffs against PRx thresholds ranging from 0.00 to 0.30 under CPP >40 mmHg conditions.

To further investigate the optimal threshold for CRx in identifying impaired cerebrovascular autoregulation, we performed threshold-based classification analyses across a range of reference PRx cutoffs (from 0.00 to 0.30, interval 0.05). The ROC analysis demonstrated that across PRx thresholds of 0.10–0.20, the optimal cutoff for CRx remained consistent at approximately −0.061, with corresponding areas under the curve (AUC) ranging from 0.849 to 0.900 (Figure 8). Sensitivity values ranged between 0.857 and 1.000, and specificity values between 0.778 and 0.800 at the identified cutoffs (Table 2). These results suggest that a CRx threshold around −0.06 provides stable and accurate discrimination of impaired autoregulation when referenced against established PRx thresholds under conditions of preserved cerebral perfusion.

**TABLE 2:** Best CRx cutoffs, AUC, sensitivity, and specificity for detecting impaired autoregulation across PRx thresholds at CPP >40 mmHg.

PRx threshold	Best CRx cutoff	AUC	Sensitivity	Specificity
0	0.01	1	1	1
0.05	0.01	0.858	1	0.667
0.1	0.06	0.849	0.857	0.778
0.15	0.06	0.9	1	0.8
0.2	0.06	0.9	1	0.8
0.25	0.09	0.897	1	0.692
0.3	0.09	0.897	1	0.692

### 3.4 CPPopt assessment from the CRx and PRx

**FIGURE 9:**
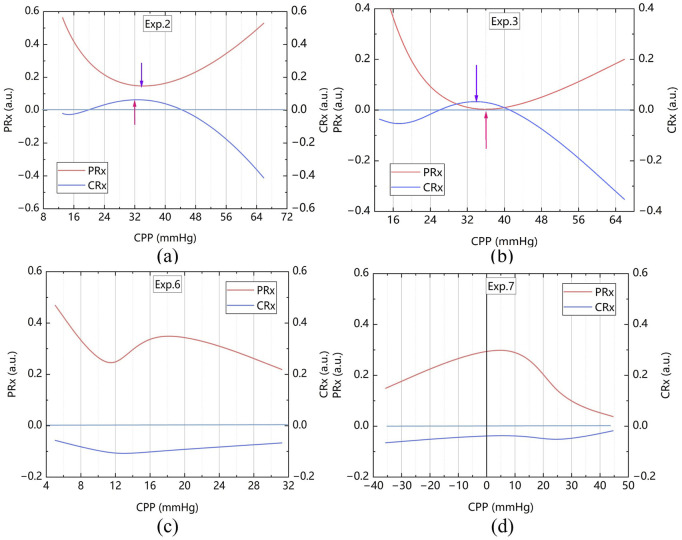
CPP-PRx and CPP-CRx Curves for determine CPPopt, smoothing performed with the Bezier algorithm **(a)** The sample No. 2 **(b)** The sample No. 3 **(c)** The sample No. 6 and **(d)** The sample No. 7.

The CPP-PRx and CPP-CRx curves were plotted, with CPPopt identified at the lowest position of the U-shaped CPP-PRx curve and the peak of the inverse U-shaped CPP-CRx curve, indicating better cerebrovascular function (Figures 9a,b). Table 3 summarizes the CPPopt results for ten rabbits. Eight rabbits exhibited identifiable CPPopt based on both PRx and CRx. However, for rabbits No. 6 and No. 7, CPPopt could not be determined due to inadequate perfusion, as shown in Figures 9c,d. This limitation was attributed to low ABP; when ICP was elevated, the CPP in both samples remained very low, particularly in sample No. 7, resulting in negative pressure.

**TABLE 3:** Comparison of optimal cerebral perfusion pressure determined by the PRx and the CRx.

Sample	CPPopt–PRx (mmHg)	CPPopt–PRx (mmHg)
No. 1	48	47
No. 2	34	32
No. 3	36	34
No. 4	32	28
No. 5	42	42
No. 6	–	–
No. 7	–	–
No. 8	47	47
No. 9	46	48
No. 10	46	44

Statistical analysis of PRx and CRx from the eight rabbits with identifiable CPPopt revealed the following results. The mean ± standard deviation of CRx at CPPopt and non-CPPopt conditions were 0.104 ± 0.170 and −0.127 ± 0.061, respectively. Similarly, the mean ± standard deviation of PRx were −0.041 ± 0.042 at CPPopt and 0.299 ± 0.082 at non-CPPopt.

**FIGURE 10:**
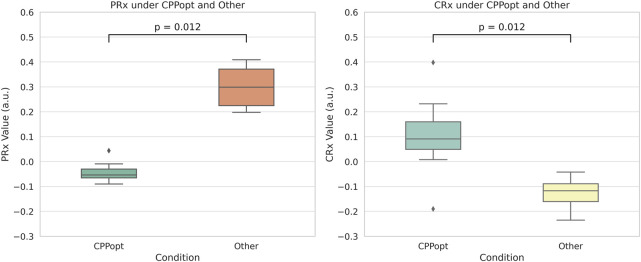
Comparison of PRx and CRx values between CPPopt and non-CPPopt conditions. Significant differences were detected using Wilcoxon signed-rank tests (p = 0.012).

Both Wilcoxon signed-rank tests and linear mixed-effects models confirm that cerebrovascular autoregulation improves significantly under CPPopt, with lower PRx and higher CRx values. As shown in Figure 10, The Wilcoxon signed-rank test showed that CRx was significantly higher at CPPopt (0.095 (0.030–0.214)) compared to non-CPPopt levels (−0.110 (−0.152–0.082)), with p = 0.012. Similarly, PRx was significantly lower at CPPopt (−0.048 (−0.074–0.012)) than at non-CPPopt levels (0.290 (0.210–0.370)), also with p = 0.012. Results from the LME were consistent with the Wilcoxon analysis. For CRx, the non-CPPopt condition was associated with a significant decrease (coefficient = −0.218, p = 0.001). For PRx, the non-CPPopt condition showed a significant increase (coefficient = +0.326, p < 0.001). These findings confirm the presence of improved cerebrovascular autoregulation under CPPopt.

To confirm that this exclusion did not bias the PRx–CRx association Excluding No.6 and 7, we calculated the PRx–CRx Correlation (n = 8). The correlation coefficient has increased to −0.513 (p = 0.001). Effect size analysis using Cohen’s q yielded q = 0.086, indicating a negligible difference (q < 0.1). Mantel–Haenszel heterogeneity test also confirmed consistency (χ^2^ = 0.01, p = 0.916), demonstrating that there was negligible difference between the PRx and the CRx Excluding No.6 and 7.

## 4 Discussion

This study evaluated the feasibility of non-invasive bio-electromagnetic detection technology in determining CPPopt and cerebral blood perfusion management by a novel bio-electromagnetic cerebrovascular function index-CRx. The animal experiment results demonstrated that CRx could determine CPPopt based on the relationship between CPP and CRx in the cerebrovascular injury rabbit model, offering a preliminary indicator for maintaining cerebrovascular function in optimal condition compared to the standard PRx.

The results revealed that CRx exhibited a significant negative correlation with PRx (r = −0.638) under low ICP conditions, indicating its potential to reflect cerebrovascular autoregulation status. However, this correlation weakened (r = −0.447) as ICP exceeded 40 mmHg, likely due to physiological differences between CRx and PRx mechanisms. This phenomenon is closely associated with the distinct physiological mechanisms influencing the two indexes. Specifically, magnetic induction phase shift (MIPS) signals underlying CRx measurements are sensitive to global changes in the brain’s electromagnetic properties, influenced by both cerebral blood volume and cerebrospinal fluid (CSF) dynamics (Xu et al., 2023). As CSF exhibits higher conductivity than blood (Gabriel et al., 1996), early ICP elevations primarily modulate MIPS through CSF redistribution. Once CSF compensation is exhausted, blood conductivity predominates, altering CRx behavior and causing divergence from PRx.

These physiological transitions are further supported by statistical analyses. Bland–Altman analysis across the full dataset revealed wide limits of agreement between CRx and PRx, and the ICC indicated poor consistency. Stratification by CPP showed that under CPP >40 mmHg—a condition where cerebral perfusion remains relatively stable and ICP is less elevated—agreement improved, with a narrower mean difference and a moderate ICC of 0.583. Although CPP was used as the stratification criterion, it is important to note that low CPP is primarily driven by elevated ICP in this animal model. These findings suggest that CRx can serve as a feasible non-invasive surrogate for PRx when ICP is not to high and cerebral perfusion is sufficient. However, the using of CRx need to be caution when interpreting under conditions of elevated ICP, where the combined effects of CSF exhaustion and effect cerebral autoregulation compromise reliability of CRx.

Threshold analysis further validated CRx’s distinguish capability of CVAR injured. Across reference PRx thresholds (0.10–0.20), a CRx threshold around −0.06 consistently achieved high diagnostic accuracy (AUC >0.85). This consistent cutoff provides a practical reference for detecting impaired autoregulation in stable CPP conditions.

Importantly, the study showed that CPPopt could be determined using CRx by plotting the CPP-CRx curve, provided that the optimal CPP fell within the measured CPP range. Among the 10 rabbits, 8 rabbits were able to determine CPPopt with the PRx, and these same 8 rabbits could also determine CPPopt with the CRx with a deviation of less than 5 mmHg compared to the PRx. Significant differences were observed between the CRx at CPPopt and the CRx at other CPP states. The PRx threshold for ABI indicated that the cerebrovascular function in the 8 rabbits was not severely injured at the optimal CPP. At this time, the CRx index was 0.104 ± 0.170 exceeded both the −0.06 and −0.08 injury thresholds, also indicating better cerebrovascular function. Both Wilcoxon signed-rank tests and LME models revealed significant improvements in cerebrovascular regulation under CPPopt. This physiological context, corroborated by low PRx values, supports the interpretation that CRx-derived CPPopt determination is valid under conditions of mild or no vascular injury. These results suggest that CRx can serve as a non-invasive alternative for CPPopt estimation and cerebrovascular perfusion management, provided that CPP is adequate. The CRx offers the potential for real-time, less invasive CVAR monitoring, particularly in settings where direct ICP monitoring is unavailable or limited.

However, when the measured CPP range did not cover the optimal CPP—such as in samples No.6 and No.7—CPPopt could not be determined, making it impossible to assess or maintain optimal vascular function. In two samples (rabbits No.6 and No.7), persistent severe hypotension (ABP <50 mmHg) led to CPP levels consistently below 20 mmHg, preventing valid curve fitting. These cases reflect limitations of the experimental CPP range rather than a flaw in the PRx–CRx methodology. The Mantel–Haenszel heterogeneity test proved that excluding these two animals did not impact the overall statistical validity. The two samples had similar features. They both had severe hypoperfusion with CPP below the ischemic threshold (<40 mmHg for rabbits), and non-U-shaped PRx–CPP patterns resembling the “unmeasurable PRx” phenomenon reported in clinical ABI cases with CVAR function failure (Steiner et al., 2002). These findings suggest two inherent limitations of CPPopt fitting: unreliable curve fitting when CPPopt falls outside the tested range, and reduced CRx sensitivity when CPP drops below 30 mmHg due to maximal vasodilation. Actually, in clinical practice, it is crucial to avoid prolonged periods of cerebral hypoperfusion, as such conditions are strongly associated with increased mortality and poor neurological outcomes in patients with ABI (Steiner et al., 2002; Needham et al., 2017). So in the further research, a stepwise norepinephrine titration protocol will be used to improve CPP of the animal model to improve model robustness under extreme physiological conditions.

Recent studies have highlighted critical limitations in the application of the pressure reactivity index (PRx) for monitoring cerebrovascular autoregulation in mild acute brain injury (mABI). PRx relies on spontaneous fluctuations of arterial blood pressure (ABP) and intracranial pressure (ICP) to assess cerebrovascular reactivity. However, in mABI patients, ICP often remains within normal limits with minimal variability, rendering PRx calculations unreliable and highly variable (Zeiler et al., 2022; Beqiri et al., 2023; Hasen et al., 2020; Motroni et al., 2024). Moreover, the invasive nature of ICP monitoring restricts the application of PRx to severe injury contexts, making it clinically impractical for the predominantly stable hemodynamics observed in mABI (Steiner et al., 2002). Non-invasive adaptations, such as NIRS-derived autoregulation indices, have been explored as alternatives. However, these modalities suffer from limited spatial resolution and susceptibility to extracranial signal contamination, particularly under low-pressure conditions (Zweifel et al., 2010; Diedler et al., 2011).

In contrast, the conductivity reactivity index (CRx), based on magnetic induction phase shift (MIPS) sensing, presents distinct advantages. Unlike PRx, CRx does not require significant ICP oscillations but instead tracks real-time changes in the brain’s electromagnetic conductivity properties related to cerebral blood volume and cerebrospinal fluid redistribution. Experimental validations have demonstrated that MIPS-based measurements can sensitively detect cerebrovascular dynamics even under stable ICP conditions, a scenario commonly encountered in mABI (Gong et al., 2024; Oziel et al., 2016). Furthermore, CRx offers fully non-invasive, continuous, and portable monitoring capabilities. These characteristics position CRx as a promising alternative for cerebrovascular autoregulation assessment in mild brain injury, where safe, dynamic, and non-invasive monitoring solutions are critically needed but currently lacking.

For the needs of whole-brain detection and subsequent multi-frequency detection, the study designed a coaxial parallel double-coil Sensor for measuring brain blood volume fluctuations and conducted experiments based on frequency at the 42 MHz. Electromagnetic simulation results demonstrated that at this frequency, the sensor exhibited high sensitivity and strong linearity in detecting changes in brain blood volume. Compared with coils optimized for specific applications, it can be used in different clinical and research environments, providing a wide range of applications. This kind of sensor has been used for multi-frequency measurements of brain electromagnetic properties, and in subsequent multi-frequency detection, it can help explore the mechanisms of cerebrovascular function through changes in various intracranial electromagnetic properties (Li G et al., 2019). However, for clinical bedside monitoring, especially targeting the depth of specific blood vessels, wearable flexible coils provide an optimal sensing solution, offering comfort and adaptability for real-time monitoring needs.

While this study focuses on single-frequency detection at 42 MHz, the multi-frequency analysis has the potential of to enhance detection robustness of CRx. Lower frequencies may offer improved separation between cerebral blood and cerebrospinal fluid (CSF) due to higher conductivity contrast, which could help in capturing compensatory responses under high ICP conditions (Li et al., 2019; Li et al., 2024). On the other hand, higher frequencies such as 65.6 MHz have been shown to be more sensitive to ischemic volume changes (Zhuang et al., 2020). Therefore, future work will explore multi-frequency fusion measurements across the 10–100 MHz range. We also aim to develop a composite CRx index that integrates amplitude, phase shift, and frequency shift features to enhance classification performance across different ICP stages.

This study has several limitations that warrant discussion. Firstly, while the potential of CRx for determining CPPopt was demonstrated through CPP-CRx curve plotting, the approach relied on invasive ICP measurements to obtain CPP. Future research should explore the use of ABP as a non-invasive substitute excitation signal for assessing cerebrovascular function in patients with mild brain injury, as suggested by the need to optimize the animal model and verify the method through larger-samples studies. Secondly, the small sample size, including two rabbits with too low CPP to determine CPPopt, indicates the need for optimizing the model. Future studies should consider adjusting CPP intervals to 5 mmHg to improve the accuracy of CPPopt determination, thereby enhancing the method’s applicability to humans.

## 5 Conclusion

This research presents a novel approach for determining CPPopt using the innovative CRx, which is derived from non-invasive magnetic induction technology. This method holds significant potential for assessing cerebrovascular function and managing cerebral perfusion pressure to optimize cerebrovascular health. The study preliminarily confirms that bioelectromagnetic technology offers a promising non-invasive alternative to PRx for cerebral blood perfusion management in patients with mild brain injuries.

## Data Availability

The raw data supporting the conclusions of this article will be made available by the authors, without undue reservation.

## References

[B100] Beqiri E. Brady K. M. Lee J. K. Donnelly J. Zeiler F. A. Czosnyka M. (2021). Lower Limit of Reactivity Assessed with PRx in an Experimental Setting. Acta Neurochir. Suppl. 131, 275–278. 10.1007/978-3-030-59436-7_51 33839857

[B101] Beqiri E. Zeiler F. A. Ercole A. Placek M. M. Tas J. Donnelly J. (2023). The lower limit of reactivity as a potential individualized cerebral perfusion pressure target: a prospective multicentre observational study. Crit. Care 27 (1), 194. 10.1186/s13054-023-04485-8 37210526 PMC10199598

[B1] Bodo M. Pearce F. J. Armonda R. A. (2004). Cerebrovascular reactivity: rat studies in rheoencephalography. Physiol. Meas. 25 (6), 1371–1384. 10.1088/0967-3334/25/6/003 15712716

[B3] Czosnyka M. Smielewski P. Kirkpatrick P. Laing R. J. Menon D. Pickard J. D. (1997). Continuous assessment of the cerebral vasomotor reactivity in head injury. Neurosurgery 41 (1), 11–19. 10.1097/00006123-199707000-00005 9218290

[B4] Dias C. Silva M. J. Pereira E. Monteiro E. Maia I. Barbosa S. (2015). Optimal cerebral perfusion pressure management at bedside: a single-center pilot study. Neurocrit Care 23 (1), 92–102. 10.1007/s12028-014-0103-8 25566826

[B5] Diedler J. Zweifel C. Budohoski K. P. Kasprowicz M. Sorrentino E. Haubrich C. (2011). The limitations of near-infrared spectroscopy to assess cerebrovascular reactivity: the role of slow frequency oscillations. Anesth. Analg. 113 (4), 849–857. 10.1213/ANE.0b013e3182285dc0 21821514

[B6] Feigin V. L. Vos T. Nichols E. Owolabi M. O. Carroll W. M. Dichgans M. (2020). The global burden of neurological disorders: translating evidence into policy. Lancet Neurol. 19 (3), 255–265. 10.1016/S1474-4422(19)30411-9 31813850 PMC9945815

[B7] Gaasch M. Schiefecker A. J. Kofler M. Beer R. Rass V. Pfausler B. (2018). Cerebral autoregulation in the prediction of delayed cerebral ischemia and clinical outcome in poor-grade aneurysmal subarachnoid hemorrhage patients. Crit. Care Med. 46 (5), 774–780. 10.1097/CCM.0000000000003016 29394184

[B8] Gabriel S. Lau R. W. Gabriel C. (1996). The dielectric properties of biological tissues: III. Parametric models for the dielectric spectrum of tissues. Phys. Med. Biol. 41 (11), 2271–2293. 10.1088/0031-9155/41/11/003 8938026

[B9] Gong Z. Zeng L. Jiang B. Zhu R. Wang J. Li M. (2024). Dynamic cerebral blood flow assessment based on electromagnetic coupling sensing and image feature analysis. Front. Bioeng. Biotechnol. 12, 1276795. 10.3389/fbioe.2024.1276795 38449677 PMC10915240

[B103] Hasen M. Gomez A. Froese L. Dian J. Raj R. Thelin E. P. (2020). Alternative continuous intracranial pressure-derived cerebrovascular reactivity metrics in traumatic brain injury: a scoping overview. Acta Neurochir (Wien) 162 (7), 1647–1662. 10.1007/s00701-020-04378-7 32385635

[B10] Hayt W. H. Buck J. (2012). Engineering electromagnetics. 8th ed. New York, NY: McGraw-Hill Education.

[B11] Hoiland R. L. Sekhon M. S. Cardim D. Wood M. D. Gooderham P. Foster D. (2020). Lack of agreement between optimal mean arterial pressure determination using pressure reactivity index versus cerebral oximetry index in hypoxic ischemic brain injury after cardiac arrest. Resuscitation 152, 184–191. 10.1016/j.resuscitation.2020.03.016 32229218

[B104] Klein S. P. Decraene B. De Sloovere V. Kempen B. Meyfroidt G. Depreitere B. (2024). The pressure reactivity index as a measure for cerebrovascular autoregulation: validation in a porcine cranial window model. Neurosurgery 95 (6), 1450–1456. 10.1227/neu.0000000000003019 38861643

[B12] Li G. Chen J. Gu S. Yang J. Chen Y. Zhao S. (2019). A dual parameter synchronous monitoring system of brain edema based on the reflection and transmission characteristics of two-port test network. IEEE Access 7, 50839–50848. 10.1109/ACCESS.2019.2911178

[B13] Li J. Zhou W. Wu L. Hu X. (2024). Noninvasive brain hemodynamics monitoring using electromagnetic coupling phase shift sensing. Front. Bioeng. Biotechnol. 12, 1276795. 38449677 10.3389/fbioe.2024.1276795 PMC10915240

[B105] Motroni V. Cucciolini G. Beqiri E. Smith C. A. Placek M. Chu K. H. (2024). Reliability and variability of pressure reactivity index (prx) during oscillatory pattern in arterial blood pressure and intracranial pressure in traumatic brain injured patients. Brain Spine 4, 102850. 10.1016/j.bas.2024.102850 39005582 PMC11246011

[B15] Needham E. McFadyen C. Newcombe V. Synnot A. J. Czosnyka M. Menon D. (2017). Cerebral perfusion pressure targets individualized to pressure-reactivity index in moderate to severe traumatic brain injury: a systematic review. J. Neurotrauma 34 (5), 963–970. 10.1089/neu.2016.4450 27246184

[B16] Oziel M. Hjouj M. Gonzalez C. A. Lavee J. Rubinsky B. (2016). Non-ionizing radiofrequency electromagnetic waves traversing the head can be used to detect cerebrovascular autoregulation responses. Sci. Rep. 6, 21667. 10.1038/srep21667 26898944 PMC4761952

[B17] Rivera-Lara L. Geocadin R. Zorrilla-Vaca A. Healy R. J. Radzik B. R. Palmisano C. (2019). Optimizing mean arterial pressure in acutely comatose patients using cerebral autoregulation multimodal monitoring with near-infrared spectroscopy. Crit. Care Med. 47 (10), 1409–1415. 10.1097/CCM.0000000000003908 31356469

[B18] Smielewski P. Czosnyka M. Zweifel C. Brady K. Hogue C. Steiner L. (2010). Multicentre experience of using ICM+ for investigations of cerebrovascular dynamics with near-infrared spectroscopy. Crit. Care 14 (Suppl. 1), P348. 10.1186/cc8580

[B19] Sorrentino E. Diedler J. Kasprowicz M. Budohoski K. P. Haubrich C. Smielewski P. (2012). Critical thresholds for cerebrovascular reactivity after traumatic brain injury: influence of hypoxia and outcome at 6 months. Crit. Care Med. 40 (6), 1970–1977. 10.1007/s12028-011-9630-8 21964774

[B20] Steiner L. A. Czosnyka M. Piechnik S. K. Smielewski P. Chatfield D. Menon D. K. (2002). Continuous monitoring of cerebrovascular pressure reactivity allows determination of optimal cerebral perfusion pressure in patients with traumatic brain injury. Crit. Care Med. 30 (4), 733–738. 10.1097/00003246-200204000-00002 11940737

[B21] Szabo S. Totka Z. Nagy-Bozsoky J. Pinter I. Bagany M. Bodo M. (2024). Rheoencephalography: a non-invasive method for neuromonitoring. J. Electr. Bioimpedance 15 (1), 10–25. 10.2478/joeb-2024-0003 38482467 PMC10936697

[B22] Tiba M. H. McCracken B. M. Ansari S. Belle A. Cummings B. C. Rajajee V. (2017). Novel noninvasive method of cerebrovascular blood volume assessment using brain bioimpedance. J. Neurotrauma 34 (20), 3089–3096. 10.1089/neu.2017.5090 28657491

[B23] Tiba M. H. McCracken B. M. Leander D. C. Colmenero Mahmood C. I. Greer N. L. Picton P. (2022). Trans-ocular brain impedance indices predict pressure reactivity index changes in a porcine model of hypotension and cerebral autoregulation perturbation. Neurocrit Care 36 (1), 139–147. 10.1007/s12028-021-01272-7 34244920

[B25] Weiss M. Albanna W. Conzen C. Megjhani M. Tas J. Seyfried K. (2022). Optimal cerebral perfusion pressure during delayed cerebral ischemia after aneurysmal subarachnoid hemorrhage. Crit. Care Med. 50 (2), 183–191. 10.1097/CCM.0000000000005396 35100191

[B26] Xu J. Li H. Jin G. Zhuang W. Bai Z. Sun J. (2023). Conductivity reactivity index for monitoring of cerebrovascular autoregulation in early cerebral ischemic rabbits. Biomed. Eng. Online 22, 78. 10.1186/s12938-023-01142-7 37559130 PMC10410901

[B27] Zeiler F. A. Aries M. Czosnyka M. Smielewski P. (2022). Cerebral autoregulation monitoring in traumatic brain injury: an overview of recent advances in personalized medicine. J. Neurotrauma 39, 1477–1494. 10.1089/neu.2022.0217 35793108

[B28] Zeiler F. A. Czosnyka M. Smielewski P. (2018). Optimal cerebral perfusion pressure via transcranial doppler in TBI: application of robotic technology. Acta Neurochir. 160 (10), 2149–2157. 10.1007/s00701-018-3687-5 30267208 PMC6209007

[B29] Zeiler F. A. Ercole A. Czosnyka M. Smielewski P. Hawryluk G. Hutchinson P. J. A (2020). Continuous cerebrovascular reactivity monitoring in moderate/severe traumatic brain injury: a narrative review of advances in neurocritical care. Br. J. Anaesth. 124 (4), 440–453. 10.1016/j.bja.2019.11.031 31983411

[B30] Zhuang W. Zhang M. Li M. Yin S. Bai Z. Sun J. (2020). A preliminary study on the feasibility of detecting global acute cerebral ischemia by the MIPS method. IEEE Access 8, 32290–32296. 10.1109/access.2020.2973250

[B31] Zweifel C. Castellani G. Czosnyka M. Helmy A. Manktelow A. Carrera E. (2010). Noninvasive monitoring of cerebrovascular reactivity with near-infrared spectroscopy in head-injured patients. J. Neurotrauma 27 (11), 1951–1958. 10.1089/neu.2010.1388 20812789

